# Working memory load improves diagnostic performance of smooth pursuit eye movement in mild traumatic brain injury patients with protracted recovery

**DOI:** 10.1038/s41598-018-36286-3

**Published:** 2019-01-22

**Authors:** Jacob L. Stubbs, Sherryse L. Corrow, Benjamin R. Kiang, Jeffrey C. Corrow, Hadley L. Pearce, Alex Y. Cheng, Jason J. S. Barton, William J. Panenka

**Affiliations:** 1 Department of Psychiatry, University of British Columbia, Vancouver, Canada; 2 Human Vision and Eye Movement Laboratory, Departments of Medicine (Neurology), Ophthalmology and Visual Science, University of British Columbia, Vancouver, Canada; 3 Department of Psychology, Bethel University, St. Paul, Minnesota USA; 4 Department of Electrical and Computer Engineering, University of British Columbia, Vancouver, Canada

## Abstract

Smooth pursuit eye movements have been investigated as a diagnostic tool for mild traumatic brain injury (mTBI). However, the degree to which smooth pursuit differentiates mTBI patients from healthy controls (i.e. its diagnostic performance) is only moderate. Our goal was to establish if simultaneous performance of smooth pursuit and a working memory task increased the diagnostic performance of pursuit metrics following mTBI. We integrated an *n*-back task with two levels of working memory load into a pursuit target, and tested single- and dual-task pursuit in mTBI patients and healthy controls. We assessed pursuit using measures of velocity accuracy, positional accuracy and positional variability. The mTBI group had higher pursuit variability than the control group in all conditions. Performing a concurrent 1-back task decreased pursuit variability for both the mTBI and control groups. Performing a concurrent 2-back task produced differential effects between the groups: Pursuit variability was significantly decreased in the control group, but not in the mTBI group. Diagnostic indices were improved when pursuit was combined with the 2-back task, and increased by 20% for the most sensitive variable. Smooth pursuit with simultaneous working memory load may be a superior diagnostic tool for mTBI than measuring smooth pursuit alone.

## Introduction

Every year, approximately 42 million individuals worldwide suffer mild traumatic brain injuries (mTBI)^1^. Repetitive brain injuries, possibly in conjunction with other genetic or environmental factors, may increase the risk for neurodegenerative diseases such as dementia^2,3^, Parkinson’s disease^4^, amyotrophic lateral sclerosis^5^, and chronic traumatic encephalopathy^6^. Diagnosing and recognizing mTBI is important for clinical decision making, prognosis, and patient safety.

At present, diagnosis largely rests upon subjective report or witness statements regarding the injury, chiefly concerning mental status symptoms such as brief loss of consciousness, confusion or disorientation, and amnesia^7,8^. Many issues surround the reliability of subjective recall in later assessments, and a diagnosis of recent mTBI is often supported by additional features, such as the presence of post-concussion symptoms and evidence of cognitive impairments on neuropsychological assessment^8,9^. However, it is not clear how sensitive such assessments are to the chronic effects of injury, and there are concerns that neuropsychological testing can be compromised by participant effort^10–12^.

The oculomotor system has recently been investigated as a potential objective marker of mTBI, as oculomotor systems use widely distributed cerebral pathways which include regions susceptible to brain injury^13^. There have been previous studies of saccades^14,15^, disconjugate eye movement^16^, and convergence dysfunction^17^, for example. Some have proposed that adding oculomotor assessments may increase the sensitivity and specificity of the current ‘gold standard’ athletic concussion instrument, the Sport Concussion Assessment Tool 5^18^, and others have proposed that the combination of complex neurocognitive tasks and eye movement recordings may provide a more sensitive marker of mTBI than either alone^13^.

One oculomotor movement of potential interest is smooth pursuit, the eye movements used to track a moving object^19^. Pursuit can be described as a sensorimotor negative feedback system that aims to reduce ‘retinal slip’ – the motion of an image on the retina – in order to maintain the object’s image near the fovea^20,21^. Previous studies have investigated smooth pursuit in mTBI, finding significant group differences between those with and without injury^22–24^. Other studies have been designed to explore how eye movements reflecting higher-level cognitive operations, such as prediction and attention, may be impaired by traumatic brain injury. Predictive anticipation of target trajectory after momentary target occlusion has been studied in patients with mTBI^25,26^. These studies have shown poorer tracking before and after target occlusion and slower resynchronization with the target upon reappearing in patients with mTBI relative to control participants^25,26^.

As neurocognitive processes such as attention are impacted following mTBI, two previous studies have evaluated the impact of divided attention on pursuit in mTBI patients, by having participants perform a word-recall task while they performed smooth pursuit tasks. One study found that pursuit variability increased when participants had to remember five-words, compared to when they had to remember one word or no words^27^. While they did not compare this to a control group, they did find that the impact of increased short-term memory load on pursuit correlated negatively with imaging measures of white matter integrity in their mTBI participants. A second study found that the pursuit of mTBI participants became more variable, compared to those without brain injury, when they had to remember five words simultaneously during pursuit^28^.

In a previous study of healthy participants, we examined the effects of working memory on smooth pursuit by having participants attend to colour changes of a pursuit target^29^. Because this attentional task involved properties of the pursuit target, it combined increases in working memory with enhanced attention to the target, rather than distracting attention from the target. Working memory load was varied with an *n*-back task, where participants had to compare the current colour of the target to the colour one change (1-back) or two changes (2-back) before. Relative to a baseline smooth pursuit task with no working memory component, these healthy participants showed decreased pursuit variability (i.e. more consistent pursuit) with the addition of the *n*-back task, demonstrating further improvements with the more challenging 2-back task than the 1-back task.

The purpose of the present study was twofold. First, we applied the same eye movement paradigm to patients who experienced mTBI and were still symptomatic, to examine whether their smooth pursuit performance changed with additional working memory load. Given that impaired attention is a common effect of traumatic brain injury, we hypothesized that these participants would be unable to show the improvement in pursuit variability seen in healthy controls. Our second goal was to characterize the diagnostic utility of this paradigm, using signal detection theory, to determine whether this provided better discrimination between healthy participants and those with mTBI.

## Methods

### Participants

We recruited patients who experienced an mTBI within the past two years, and were symptomatic at the time of testing. We also recruited healthy controls matched on age (±five years) and gender, who denied ever having an mTBI. Patients and controls were recruited through an electronic newsletter distributed by the local health authority. A power analysis using G*Power 3.1 was conducted based on our previous study of healthy participants and existing literature examining smooth pursuit eye movement deficits in mTBI^29,30^. The analysis (α = 0.05, power = 0.8, and desired effect size of 1.3 commensurate with our previous results and the existing literature) projected that a sample size of 10 participants would be necessary in each group for the simplest between-group comparison. Thus, we aimed to recruit a sample of 16 participants per group to allow for potential attrition and conservative estimate.

mTBI was diagnosed with the Ohio State Identification method using the diagnostic criteria of the World Health Organization^31,32^. Age limits for this study were 18–50 years, the lower limit representing the age at which visual pursuit is thought to be fully developed, and the upper limit representing a conservative limit after which age-related declines in pursuit may occur^33^. Visual acuity for all participants was normal (defined as 20/20) or corrected-to-normal with glasses or contacts. Participants were allowed to wear glasses or contacts if needed, and calibration was performed with lenses in place. Participants were pre-screened prior to enrollment, and were excluded if they had a history of eye or vision disorders, neurological or psychiatric illness, a present or past history of drug or alcohol abuse, or, if at the time of testing, they were using a psychotropic medication or nicotine, had cranial nerve abnormalities, or were pregnant. One control was excluded from analyses for repeatedly failing to follow task instructions. This protocol was approved by the Clinical Research Ethics Boards of Vancouver General Hospital and the University of British Columbia, and all experiments were conducted in accordance with the relevant guidelines and regulations. Informed consent was obtained from all participants in accordance with the Declaration of Helsinki.

### Apparatus and stimuli

Eye movements were recorded monocularly at 500 Hz using an EyeLink 1000 system with tower mount (SR Research, Canada). Stimuli were presented using an in-house developed python script, on a ViewSonic VX2268wm monitor (ViewSonic Corp. USA) with a refresh rate of 120 Hz, placed 60 cm away from the participant. Monitor resolution was set at 800 × 600 pixels, and the graphics card used for the stimuli was a 128-bit PNY Quadro K620 (PNY Technologies Inc., USA). Reaction time measurements were recorded using a Logitech F310 gamepad (Logitech International S.A., Switzerland).

The target was a white ring subtending 0.5° of visual angle with an inner disc having a diameter of 0.25° of visual angle, displayed on a dark grey background (RBG colour code 75 75 75). On stationary blocks (i.e. baseline *n*-back) the target appeared at the centre of the screen. On pursuit blocks the target moved clockwise around a circular trajectory with a radius subtending 10.5° of visual angle, at 0.4 Hz centered on the screen.

The secondary *n*-back task involved attention to changes in the colour of the central disc of the target. One of nine colours appeared in the central disc in a pseudo-random order. During the 1-back task, the disc alternated between one of the nine colours (1000 ms) and the background grey (1000 ms). During the 2-back task, the central disc alternated between one of the nine colours every 1000 ms. For the 1-back task, participants were instructed to press a key when a colour was repeated sequentially (e.g. blue-grey-yellow-grey-orange-grey-**orange**; where bolded text indicates a correct key-press). For the 2-back task they were instructed to press the key if the colour matched that presented two colours previously (e.g. orange-blue-red-green-**red**…).

### Procedure

Nine-point calibration and validation procedures were performed before each task, and were redone if the average margin of error exceeded 0.5° of visual angle. Participants first performed a baseline *n*-back task while watching a stationary target. They then performed a baseline pursuit assessment (i.e. without the n-back task), with instructions to simply follow the target with their eyes around its trajectory as smoothly as possible. Finally, they performed the dual-task condition, with simultaneous smooth pursuit and *n*-back task. Each of these three components lasted 60 seconds each. They then repeated the process for the other *n*-back task. Half of the participants were randomized to perform the 1-back block first and the 2-back block second, while the remaining half performed the blocks in the reverse order. At the 30-second mark of each pursuit trial, a drift correction was performed to compensate for any inadvertent signal drift during the trial. Participants were allowed to take a short break between each component to minimize fatigue. Participants then filled out demographics and injury questionnaires, as well as the Rivermead Post-Concussion Symptoms Questionnaire^34^.

### Data variables

The same analysis was used in our previous study^29^, and further explanations are available in Supplementary Methods. Means and standard deviations for reaction times were calculated, and due to each colour change lasting a maximum of 1000 ms, we excluded reactions times above 1000 ms for the purposes of analysis (which comprised only 5.3% of all possible responses). Missed and false cues were recorded, and the sum of these errors is reported as the ‘error rate’.

For the pursuit blocks, the EyeLink parser was used to detect blinks and saccades. Saccades were defined as eye movement velocity above 35°/sec or acceleration above 9500°/sec^2^, which provided a good threshold for separating pursuit from corrective saccades, given that the tangential velocity of the target was approximately 26°/sec. Saccades were excised from the data prior to analysis. The remaining eye movement data were analyzed using an in-house developed MATLAB script (The MathWorks, USA). We excluded the first full cycle of each pursuit block so that our analysis focused on the steady-state pursuit that followed initiation of pursuit.

We first characterized pursuit performance for the remaining trace with traditional one-dimensional (horizontal and vertical) measures of gain and phase. We used a cross-correlation procedure that varied the offset between eye and target velocity to find the offset that gave the smallest residual error. The mean offset was termed *phase offset*, expressed in degrees per cycle, and is a metric of phase lead or lag. Once phase offset was corrected, *gain* was defined as the slope of the linear regression of eye velocity over target velocity. Further explanation of phase offset and gain are reported in Supplementary Methods Figures S1–S3.

We next performed a two-dimensional analysis, also employed in our previous study and by others^29,35,36^, which assessed positional errors after converting the data to polar coordinates. For each sample along the target trajectory, we calculated measures of spatial error ahead and behind, and inside and outside the target trajectory, respectively. *Tangential error* was the vector between the eye and target position as projected onto the direction of instantaneous target motion (quantifying gaze position lead or lag.) Positive values indicate gaze position ahead of the target. *Radial error* was the vector orthogonal to instantaneous target motion, with a positive value indicating an eye position further away from screen center than the target. Both tangential and radial errors were expressed in degrees of visual angle. For each of these two position variables, we calculated the standard deviations as indices of the variability of pursuit, where smaller values would indicate more consistent pursuit. Because standard deviations were not normally distributed, we applied a log_10_ transform to those data to create the variables *tangential variability* and *radial variability*.

Finally, to capture positional variability in a single metric, we multiplied the standard deviation of radial error by that of tangential error, and then by π – a variable that can be visualized as the area of an ellipse – and we termed the log_10_ transform of this value *overall variability*. Further explanation of the two-dimensional variables is provided in Supplementary Methods Figures S4 and S5.

### Statistical analysis

For the participant’s key press responses to the change in target colour, we analyzed the variables of reaction time and error rate using a general linear model, with main factors of participant group (control, mTBI), task (1-back, 2-back), and ocular motor condition (stationary, pursuit), with participant as a random factor. All reaction times satisfied the assumption of normality as assessed by the Shapiro-Wilk test (p > 0.05).

For the pursuit variables, to simplify the analysis, we collapsed the baseline conditions (pursuit without an *n*-back task), as our prior analysis had shown no significant difference between these^29^. Thus, we analyzed the pursuit variables with a general linear model with two main factors, participant group (control, mTBI), and working memory condition (baseline, 1-back task, 2-back task) with participant as a random factor. Significant effects were explored *post hoc* with Tukey’s honest significant difference (HSD) test.

For the signal detection theory analysis, we used the means and standard deviations from the log_10_ transformed data to construct binomial receiver operator characteristic curves for radial variability and overall variability, for each of the baseline, 1-back and 2-back pursuit conditions^37^. When the data are z-transformed, the curves become linear, and can be characterized by their slope *s*. Because these slopes differed from 1, which occurs when the standard deviations of the scores of the two groups differ, we computed the discriminative index *d*_*a*_’. For each variable we also calculated the specificity when we set a criterion that yields a sensitivity of 95%, which we call the *screening specificity*. This reveals how well each task performs as a screening test. Conversely, to determine utility as a diagnostic instrument, we calculated the sensitivity when specificity is set to 95%, which we termed *diagnostic sensitivity*.

## Results

The mTBI (*n* = 16) and control (*n* = 15) groups did not differ significantly in age (t_(29)_ = 0.094, p = 0.926), or gender (χ^2^ = 1.136, p = 0.567). Post-concussion symptoms were significantly higher in the mTBI group (p < 0.0001) than controls. The mean time since injury was 5.2 months (SD = 3.8 months; range = 0.6–13 months). All demographics and injury characteristics are reported in Table 1.

**Table 1:** Participant demographics and mTBI group injury characteristics.

Characteristic	mTBI (n = 16)	Control (n = 15)	p =
Age, mean (SD)	31.4 (6.3)	31.1 (8.0)	0.926
Gender, n females (%)	13 (81.3%)	13 (86.7%)	0.567
Months post-injury, mean (SD)	5.2 (3.8)	N/A	
Rivermead score, mean (SD)	33.31 (11.44)	2.33 (3.45)	<0.0001
Mechanism of Injury (%)			
Vehicle accident	31.3%	N/A	—
Sport	43.8%	N/A	—
Fall	25.0%	N/A	—
Other	6.3%	N/A	—
Hospital Visit for Injury	25.0%	N/A	—
Loss of consciousness (%)			
Yes	12.50%	N/A	—
No	75.00%	N/A	—
Unknown	12.50%	N/A	—
Post-traumatic amnesia (%)	12.50%	N/A	—
Eligible for compensation (%)	31.25%	N/A	—
Number of prior head injuries, mean (SD)	1.75 (2.1)	N/A	—

P-values denote the significance value obtained through an independent samples t-test for age and Rivermead score, and Chi-square test for gender.

### *N*-back task performance

**Figure 1:**
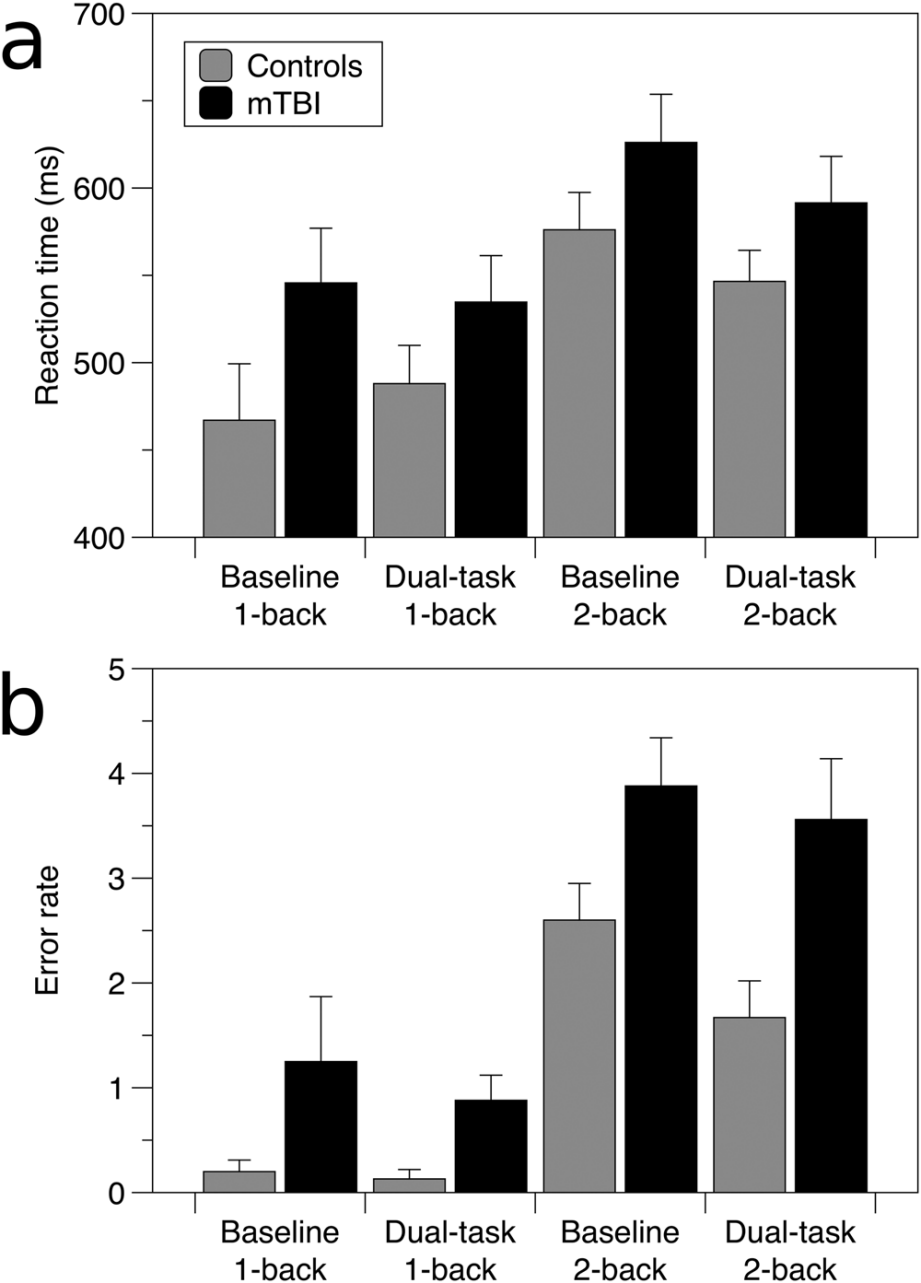
Working memory task results. Reaction times for the n-back tasks at baseline and during pursuit (dual-task). (**a**) Reaction time, and (**b**) error rate for the control (grey) and mTBI groups (black). Error bars represent one standard error.

For reaction time (Fig. 1a), there was a trend to an effect of group, with control participants faster on average than the mTBI participants, however this was not statistically significant (F_(1,29)_ = 4.04, p = 0.054). There was an effect of task (F_(1,87)_ = 28.4, p < 0.0001), with responses faster during the 1-back than the 2-back task for both groups. There was no effect of ocular motor condition, and no significant interactions. The results for error rate were similar (Fig. 1b): a main effect of group, with controls making significantly fewer errors than the mTBI group (F_(1,29)_ = 12.8, p < 0.002); an effect of task (F_(1,87)_ = 76.7, p < 0.0001), with more errors in the 2-back than the 1-back condition for both groups, and no effect of ocular motor condition or significant interactions.

### Eye movement data

#### Traditional one-dimensional Cartesian analyses

**Figure 2:**
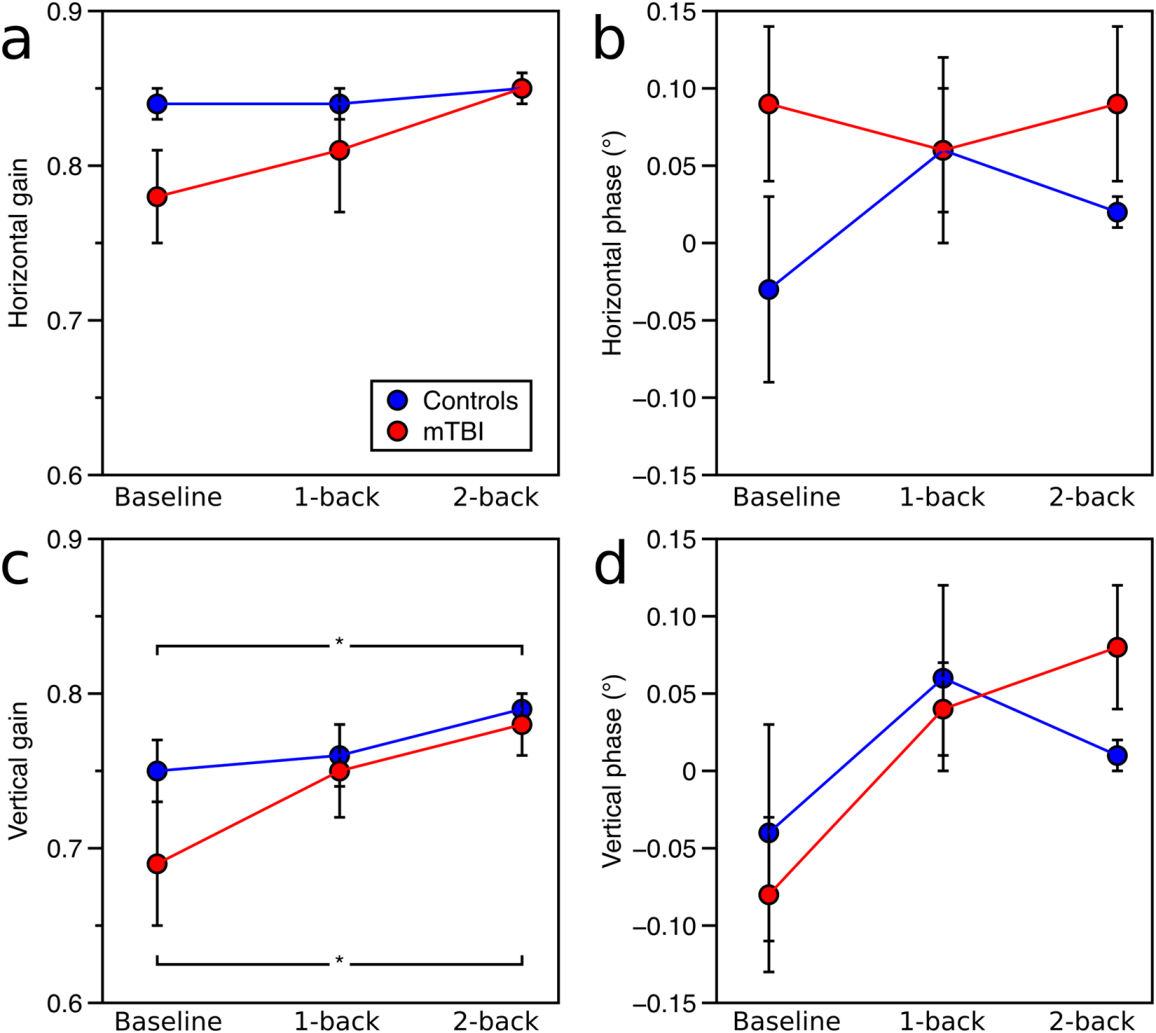
One-dimensional smooth pursuit results. (**a**) Horizontal vector gain, (**b**) horizontal phase offset, (**c**) vertical vector gain, and (**d**) vertical phase offset across conditions for the mTBI group (red) and controls (blue). Error bars represent one standard error.

For horizontal gain, there was slightly lower gain in the mTBI group, though this was not significant, (F_(1,29)_ = 3.10, p = 0.089), and there was no effect of working memory condition or interaction (Fig. 2a). Horizontal phase showed no effects, Fig. 2b.

For vertical gain, there was no effect of group but there was an effect of working memory condition (F_(2,58)_ = 4.55, p = 0.015), with slightly higher gain with the 2-back task than baseline condition for both groups (p < 0.05; Fig. 2c). There was no significant interaction. Vertical phase showed a slight effect of working-memory condition (F_(2,58)_ = 3.19, p = 0.049), though there were no significant differences in pair-wise contrasts (Fig. 2d).

#### Two-dimensional analyses

**Figure 3:**
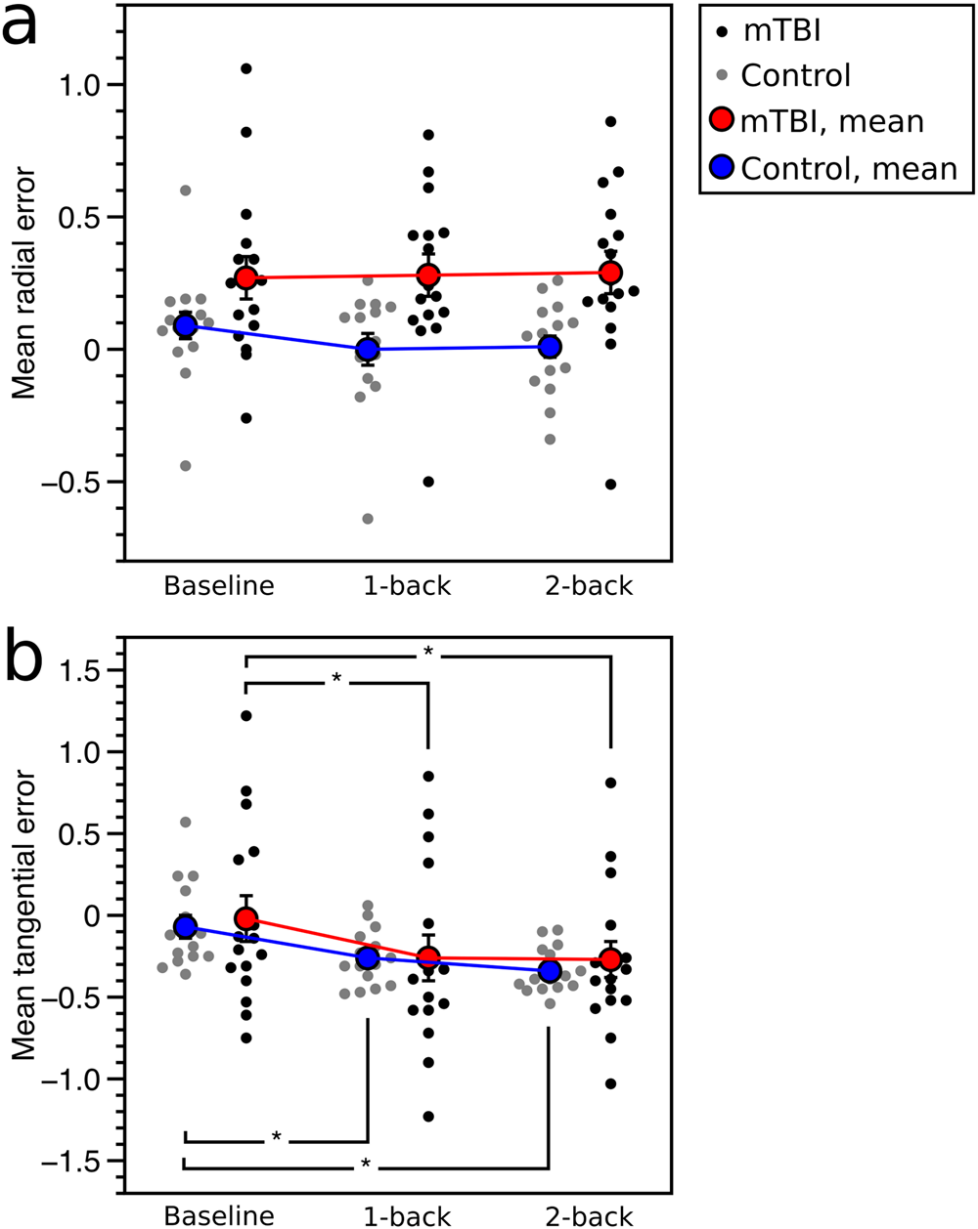
Two-dimensional smooth pursuit results for mean radial and tangential error. (**a**) Mean radial error, and (**b**) mean tangential error, for mTBI patients (black: individual patients; red: group mean) and controls (grey: individual controls; blue: group mean). ‘*’ denotes p < 0.05 for Tukey’s HSD, and error bars represent one standard error.

Radial and tangential mean error: Mean radial error showed an effect of group (F_(1,29)_ = 8.63, p = 0.006) with more error in the mTBI group, but no effect of working-memory condition or interaction (Fig. 3a). Mean tangential error showed no effect of group but an effect of working memory condition (F_(2,58)_ = 28.83, p < 0.0001; Fig. 3b), with more negative error in the 1-back and 2-back-tasks compared to baseline for both groups (p < 0.05). There was no interaction.

**Figure 4:**
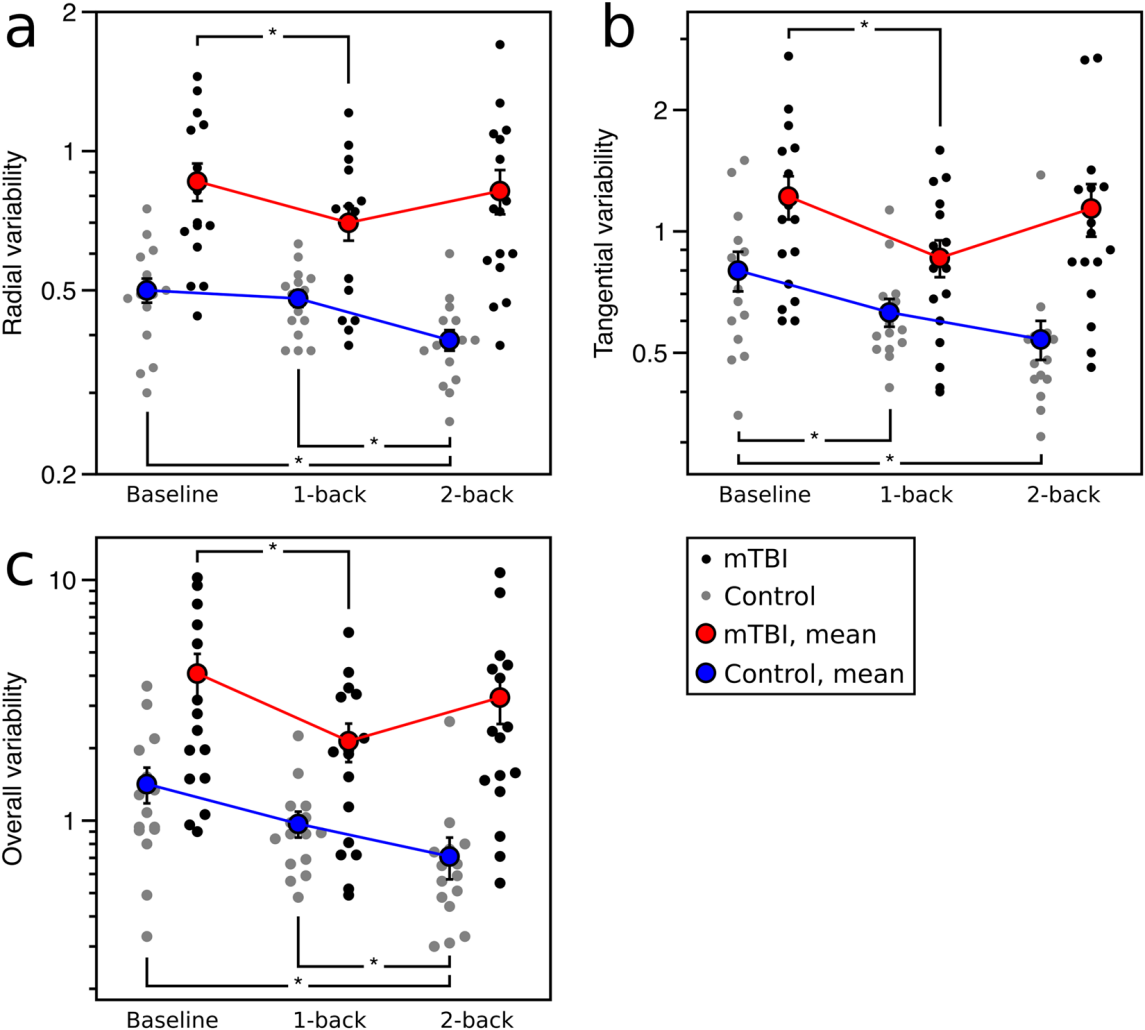
Two-dimensional smooth pursuit results for radial, tangential, and overall variability. (**a**) Radial variability, (**b**) tangential variability, and (c) overall variability for mTBI patients (black: individual patients; red: group mean) and controls (grey: individual controls; blue: group mean) across each condition. ‘*’ Denotes p < 0.05 for Tukey’s HSD, and error bars represent one standard error.

Radial and tangential variability: Radial variability showed an effect of group (F_(1,29)_ = 31.1, p < 0.0001), with the mTBI group showing more variable pursuit (Fig. 4a), and an interaction between group and working memory condition (F_(2,58)_ = 4.71, p = 0.013). There was a trend to an effect of working-memory condition (F_(2,58)_ = 3.10, p = 0.053), with *post hoc* contrasts showing less variability in the 2-back-task than during the baseline and 1-back conditions for controls (p < 0.05), and less variability in the 1-back condition than baseline for mTBI patients.

Tangential variability showed an effect of group (F_(1,29)_ = 12.43, p = 0.0014), with more variable pursuit in the mTBI group (Fig. 4b), and an interaction between group and working memory condition (F_(2,58)_ = 5.40, p = 0.007). There was an effect of working-memory condition (F_(2,58)_ = 7.07, p = 0.002), with *post hoc* contrasts showing less variability in the 1-back and 2-back conditions compared to baseline for controls, and less variability in the 1-back condition than baseline for mTBI participants (p < 0.05).

Overall variability: There was more overall variability in the mTBI group (F_(1,29)_ = 19.22, p < 0.0001; Fig. 4c), and a significant interaction between group and working-memory condition (F_(2,58)_ = 5.75, p = 0.005). *Post-hoc* contrasts showed that for the control group, the 2-back condition resulted in significantly less pursuit variability than in the baseline and 1-back conditions, while the mTBI group had less variability in the 1-back task than the baseline condition.

Finally, we performed bivariate correlations to examine whether there was a relationship between time since injury in the mTBI group, and all eye movement metrics. There was a significant negative correlation between mean radial error and time since injury (r = −0.60, p = 0.013), however this association was not significant in either the baseline or 2-back conditions. No other pursuit metrics were significantly associated with time since injury. However, caution is required in the interpretation of the significance of these correlations due to the modest sample size.

**Figure 5:**
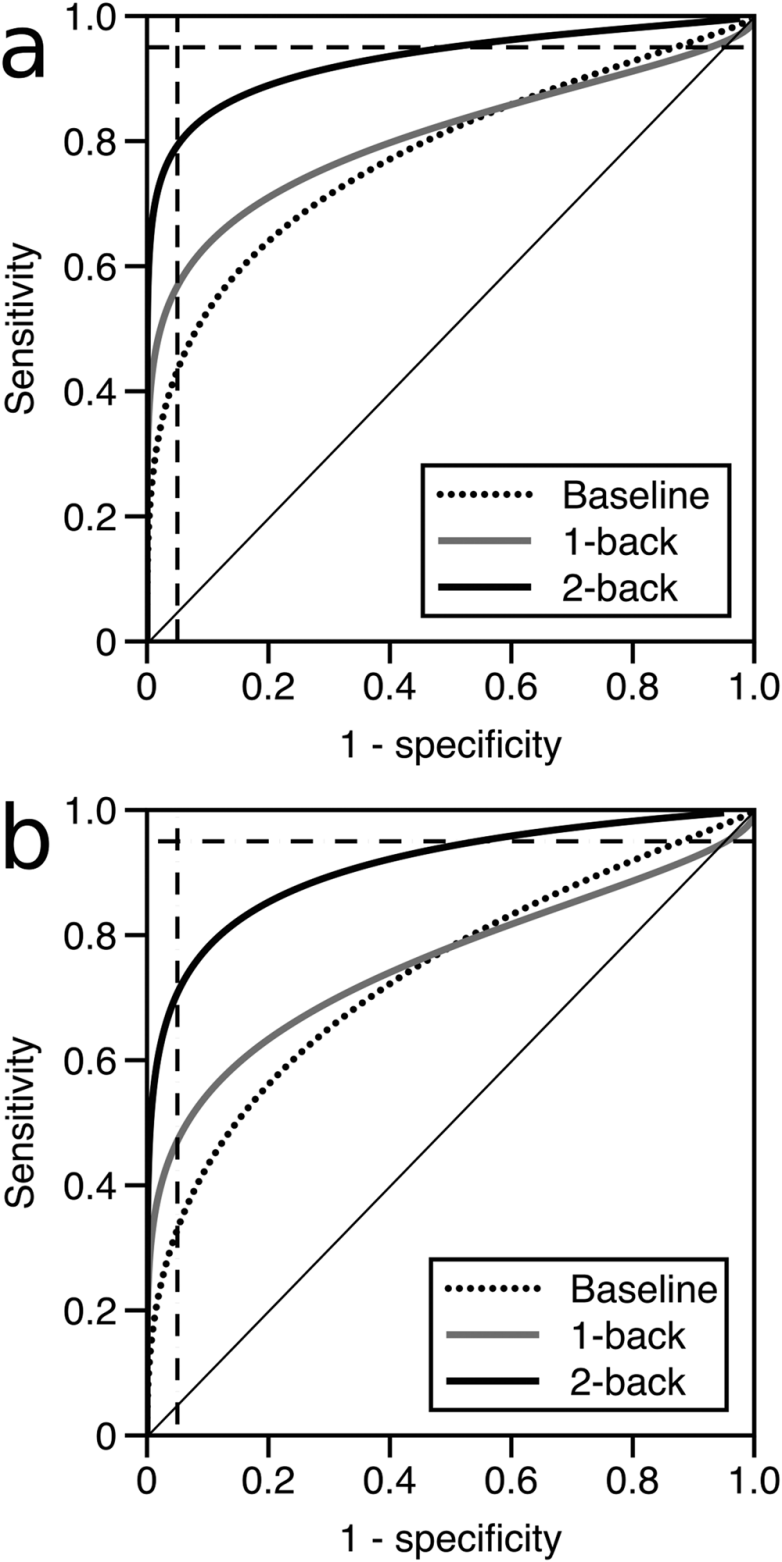
Binomial receiver operator characteristic curves (ROC) for metrics of smooth pursuit variability. (**a**) Radial variability ROC, and (**b**) overall variability ROC (dotted curve: baseline pursuit; grey curve: pursuit with simultaneous 1-back task; black curve: pursuit with simultaneous 2-back task). Dashed lines denote cutoff values to derive the ‘diagnostic sensitivity’ and ‘screening specificity’ variables.

Signal detection theory analysis of diagnostic performance: Figure 4 shows that the best variables to separate the mTBI and control groups are radial variability and overall variability, and we explored the diagnostic utility of these two measures in discriminating between healthy control and mTBI participants. The area under the curve of an empiric receiver operator characteristic (AUC), discriminative index (*d*_*a*_’), and our variables of diagnostic sensitivity and screening specificity all showed the highest diagnostic performance during smooth pursuit with the concurrent 2-back task (Table 2). The analysis also shows that both tests work better as diagnostic rather than screening tests. For the diagnostic sensitivity of baseline pursuit (i.e. with a 95% specificity), mTBI participants would be classified correctly 58% of the time on radial variability and 44% of the time on overall variability, see Fig. 5a,b. The diagnostic sensitivity was improved by approximately 20% with a concurrent 2-back task when compared to baseline pursuit, as pursuit would correctly classify 79% of patients using radial variability or 71% of patients with overall variability, in the dual-task condition with 95% specificity.

**Table 2:** Diagnostic performance analysis of radial and overall smooth pursuit variability.

Radial variability	AUC	*d*_*a*_'	Diagnostic sensitivity	Screening specificity
Baseline	0.879	1.61	0.58	0.38
1-back	0.783	1.22	0.57	0.07
2-back	0.946	2.08	0.79	0.50
**Overall variability**				
Baseline	0.796	1.21	0.44	0.21
1-back	0.717	0.97	0.47	0.05
2-back	0.904	1.89	0.71	0.46

AUC is the area under the curve for an empiric receiver operator characteristic; d_a_’ is the signal detection theory discriminative index; diagnostic sensitivity is the sensitivity at 95% specificity, and screening specificity is the specificity at 95% sensitivity, derived from binomial receiver operator characteristics.

## Discussion

We evaluated the diagnostic performance of smooth pursuit eye movement with concurrent working memory load in mTBI patients and healthy controls. We chose an *n*-back task to load working memory as it is largely independent of stimulus type^38^, and we were able to integrate it directly into the pursuit target. The 2-back condition was more challenging than the 1-back condition – as evidenced by longer reaction times and increased error rate in the 2-back task – however, there were no degradative effects of pursuit on *n*-back performance in healthy controls^29^. In the current study, mTBI participants had more difficulty performing the *n*-back tasks than healthy participants, with more errors and longer reaction times, but similarly did not show any degradation in pursuit with increased working memory load.

Our one-dimensional analysis showed only a trend to lower gain in the horizontal plane after mTBI. This suggests that the basic ocular motor operations generating a pursuit response are intact in the mTBI group. In the two-dimensional analysis, mTBI participants showed increased mean radial error, with mTBI participants erring outside the target trajectory on average. However, the primary abnormality shown by the mTBI patients was a larger variability of pursuit, with greater variability in the radial and tangential vectors.

In our report on healthy participants, we found that the addition of a 1-back task reduced pursuit variability, which was further reduced with the more difficult 2-back task^29^. In our mTBI participants, there was also a reduction in variability when attention was enhanced towards the target with the addition of the 1-back task during pursuit. However, there was no such improvement in variability with the concurrent 2-back task, when compared with baseline. Hence, this inability of the mTBI group to improve pursuit variability further with the 2-back task suggests a limitation of attentional capacity following mTBI. This is supported by our signal detection theory analysis showing that pursuit with the 2-back condition yielded the best diagnostic performance in distinguishing between mTBI and control participants.

In addition to the preserved effects of focused attention in the 1-back task, another effect of attention was preserved in our mTBI participants, as shown in the analysis of mean tangential error. In our previous report on healthy participants, we found that adding the working memory task resulted in a more negative mean tangential error – in other words, an increased phase lag^29^. Given evidence that the focus of attention during pursuit lies 1° to 3° in front of current eye position^39,40^, we speculated that increased phase lag may serve to place attention on the target. In the current study, we found that both healthy and mTBI participants showed an increase in phase lag with the *n*-back tasks.

While the mTBI group showed preservation of some attentional effects, such as generation of a phase lag and more consistent pursuit with the focused attention and working memory required during the 1-back task, the key difference was higher pursuit variability with the more attentionally demanding 2-back task. This deleterious effect on variability parallels other findings of greater variability of performance after mTBI^41,42^, or with other disorders of attention on a variety of tasks such as reaction time and motor-timing^43^. It is also consistent with studies in healthy participants that find more visuomotor tracking error during lapses of attention or ‘mind-wandering’^44^.

Our results can be compared to some extent with those found in a study that examined pursuit with and without a concurrent one or five-word recall task^28^. Contreras and colleagues (2011) found that pursuit variability was increased in mTBI participants when they had to remember five words concurrently, as compared with one or no words, and no significant differences were found in the control group. Such a task can be considered to distract or divide attention away from pursuit, whereas our task focused attention towards the pursuit target. This may account for why, with the less difficult 1-back task, we found decreased pursuit variability for both groups while they did not. However, there are similarities in the effects of increased attentional load. As reflected in their mean or median synchronization indices, pursuit variability did not differ greatly between healthy and mTBI participants with either no task or the need to remember one word, but variability was significantly greater in the mTBI group than in the control group when having to remember five words. Similarly, we found that the pursuit performance of our mTBI group differed more from that of the controls with the more challenging 2-back than the 1-back task.

Higher values of *n* invoke a variety of complex cognitive processes including temporal encoding, rehearsal, inhibition, and interference resolution^45^. Our results are analogous to neuroimaging studies that have found that mTBI patients and controls show differential effects of increasing *n* on neural activation patterns during *n*-back tasks. Neural activation in bilateral parietal and frontal regions has been shown to increase in a step-wise pattern for increasing values of *n* in controls, whereas mTBI patients show an exaggerated increase for moderate load and then plateau for larger values of *n*^46,47^. This disproportionate neural activation as well as the combination of acceptable accuracy with slower response times of patients with mTBI has been interpreted as an indication of reduced cognitive efficiency^48^.

The diagnostic utility of smooth pursuit in mTBI has seldom been assessed in previous studies, yet represents an important clinical consideration. One study of a similar circular pursuit task reported an AUC (area under the curve) of 0.86 for the ability of either tangential or radial variability to discriminate patients with mTBI from healthy participants^49^. We replicated this finding for radial variability in the baseline condition, with an AUC of 0.88, and found a higher AUC of 0.946 when pursuit was combined with a concurrent 2-back task. Similarly, for our metric of overall variability, while the baseline AUC was initially at 0.80, it was improved to 0.90 with the integration of the 2-back task. In our examination of diagnostic performance, all variables performed better as diagnostic rather than screening instruments, which can be attributed to the larger variance of the mTBI group compared to the controls. The diagnostic performance of pursuit was improved with a concurrent 2-back task by decreasing pursuit variability in the control group while the mTBI group was unable to manifest similar improvement. Of the variables analyzed, radial variability during the 2-back condition showed the best discriminative ability, with *d*_*a*_’ of 2.08. As a diagnostic test with 95% specificity, baseline radial variability correctly identified 58% of patients, which increased by more than 20% with the concurrent 2-back task to classify 79% of patients as abnormal.

The present study is not without its limitations. Detailed neuropsychological testing was not done, and it would be of interest for future studies to compare the ocular motor results to traditional measures of working memory, attention and executive function. Indeed, it may be that these are closely related, and that our ocular motor paradigm offers a rapid and fairly accurate means of characterizing such deficits. We also did not characterize our group for other effects such as convergence insufficiency, dizziness, anxiety or depression, and whether these would also show a relationship is not known. However, previous research indicates that there may be no interaction between depression or anxiety and smooth pursuit^50^. We did not evaluate for potential effects of prebyopia, as there were only three patients over the age of 35, thus an age-stratified analysis would not have sufficient power. Moreover, the paradigm does not require high spatial resolution to perceive the target location or colour. Our sample size was modest, though similar to other studies in this population^28,36,49^, thus, further study to replicate these finding in a larger sample and to determine the relationship of the results to other neuropsychological measures is warranted.

In summary, we present evidence for some aspects of preserved attentional effects in pursuit tasks after mTBI. Both mTBI and control participants showed reduced pursuit variability when attention to the target of pursuit was enhanced with a 1-back task, and both groups showed increase in phase lag with *n*-back tasks, which we speculate may serve to focus attention better on the target. However, when the attentional task became more difficult, pursuit variability was further reduced in the control group but not in the mTBI group, indicating a limitation of attentional capacity in the latter. The result was greater diagnostic separability between the controls and mTBI group, compared to the baseline pursuit task where an *n*-back task was not performed. Our analysis of diagnostic performance suggested that, as a diagnostic test, pursuit variability could correctly classify approximately 20% more patients with mTBI as having an abnormality if smooth pursuit was performed with a concurrent 2-back task rather than without it.

## Electronic supplementary material

Supplementary methods

## Data Availability

The datasets generated during the current study are available from the corresponding author on reasonable request.
