# Supplementary material for: Working memory load improves diagnostic performance of smooth pursuit eye movement in mild traumatic brain injury patients with protracted recovery
Source: Sci Rep. 2019 Jan 22;9:291. doi: 10.1038/s41598-018-36286-3 (PMC6342945; doi:10.1038/s41598-018-36286-3)
Supplement: Supplementary file 1 — Supplementary methods [file 41598_2018_36286_MOESM1_ESM.pdf]

Supplementary Methods for article in *Scientific Reports*

Title: Working memory load improves diagnostic performance of smooth pursuit eye movement in mild traumatic brain injury patients with protracted recovery

Authors: Stubbs, J. L., Corrow, S. L., Kiang, B. R., Corrow, J. C., Pearce, H. L., Cheng, A. Y., Barton J. J. S., Panenka W. J.

Page 2-3: Phase offset
Page 4: Gain
Page 5-6: Two-dimensional variables

Phase offset: We analyzed horizontal and vertical pursuit separately, thus deriving two sinusoids from the circular pursuit trace. For both horizontal and vertical pursuit, we varied the gaze velocity trace (Figure 1, blue) forward and backwards in time, to find the lowest offset between the gaze and target velocity traces (black) to attempt to correct any offset in phase (red). The mean amount that we moved the trace forwards or backwards, quantified in degrees per cycle (i.e. degrees around the circle), is termed 'phase offset'. Phase offset can be visualized as a function of time, Figure 2, allowing easier conceptualization of mean phase offset.

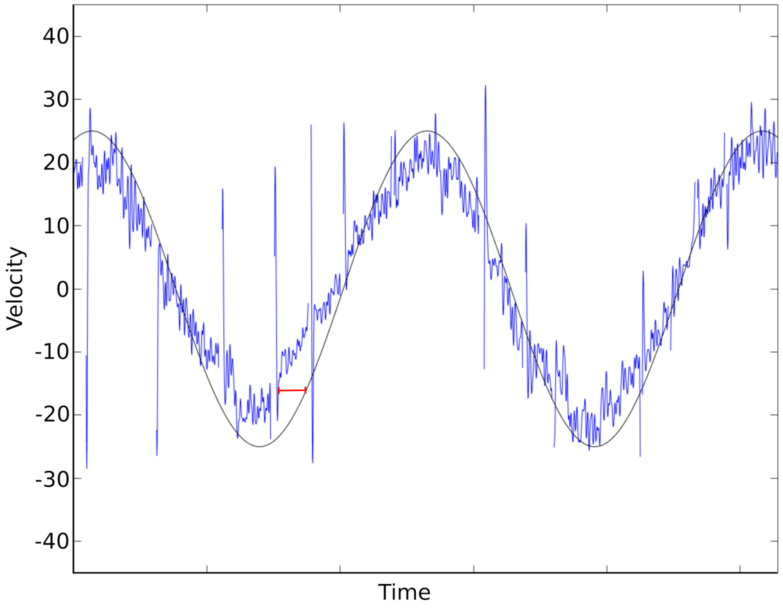

Figure S1 | **Raw data used to drive horizontal phase offset**. Horizontal gaze velocity (blue) and target velocity (black) as a function of time, with phase offset denoted in red.

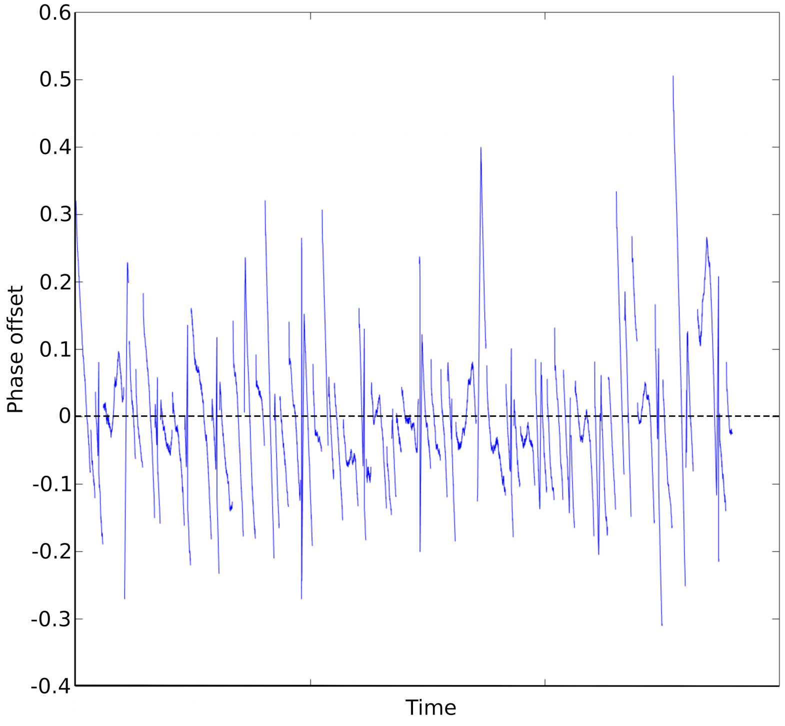

Figure S2 | **Horizontal phase offset as a function of time**. Positive values denote phase lead, and negative values denote velocity behind the target (i.e. phase lag).

Gain: To calculate gain, we plotted gaze velocity over target velocity (blue), and fitted a linear regression line (Figure 3). The slope of the regression line represents gain.

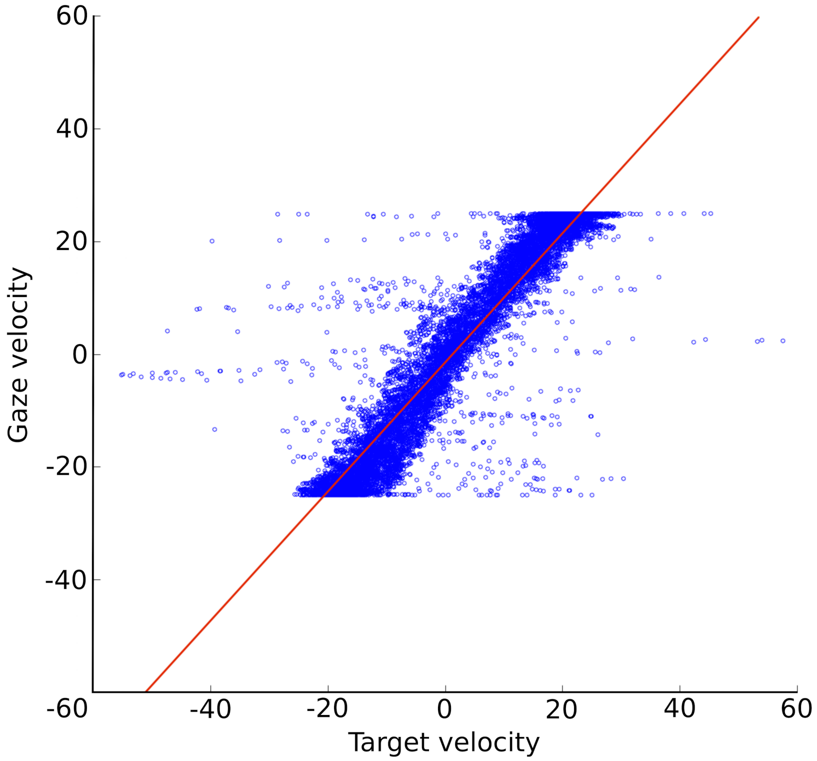

Figure S3 | **Scatter plot to derive gain**. Gaze velocity plotted over target velocity for each sample in the pursuit trace (blue) with a fitted linear regression line (red).

Two-dimensional variables: The raw positional data for the gaze and target can be visualized, Figure 4. For each sample (target position and gaze position at each point in time), we rotated the target and associated gaze positions to overlay all target positions, to produce Figure 5. Any error ahead or behind the direction of target movement is tangential error, where we can find 'mean tangential error' (quantifying positional phase lead or lag) as well as the standard deviation of tangential error, which we termed 'tangential variability'. Error inside or outside the target trajectory is radial error, again, where we can find the 'mean radial error', or, using the standard deviation of radial error, 'radial variability'.

Multiplying tangential variability, radial variability, and π results in the shaded elliptical area in Figure 5, which we termed 'overall variability'.

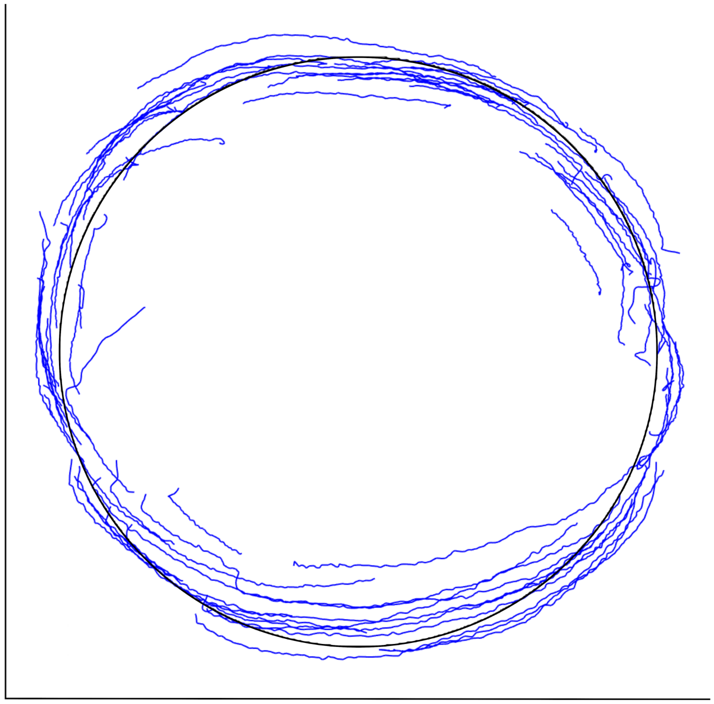

Figure S4 | **Raw pursuit data**. 30-second trace of gaze position (blue) and target position (black).

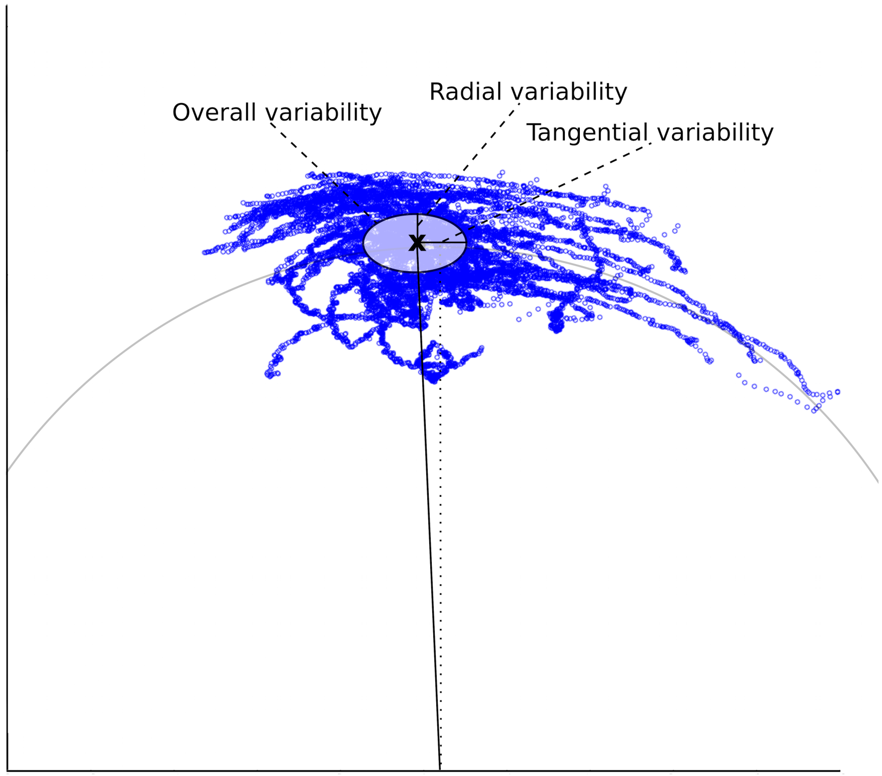

Figure S5 | **Two-dimensional variables**. Tangential error represents gaze position ahead or behind of the target position, and radial error represents gaze position inside or outside of the target trajectory. Overall variability is a composite measure quantifying positional error in a single metric.
